# Supplementary material for: The selection of software and database for metagenomics sequence analysis impacts the outcome of microbial profiling and pathogen detection
Source: PLoS One. 2023 Apr 7;18(4):e0284031. doi: 10.1371/journal.pone.0284031 (PMC10081788; doi:10.1371/journal.pone.0284031)

**Fig S1.** The number of distinct taxa identified by profiles classified by different software and DBs. The red regression line was fitted with the profiles classified by all software and DBs. The black regression line was fitted using all profiles excluding the one by Diamond+Megan and Metaphlan3. R^2^ values for each fitted line were labelled with the corresponding color.

**
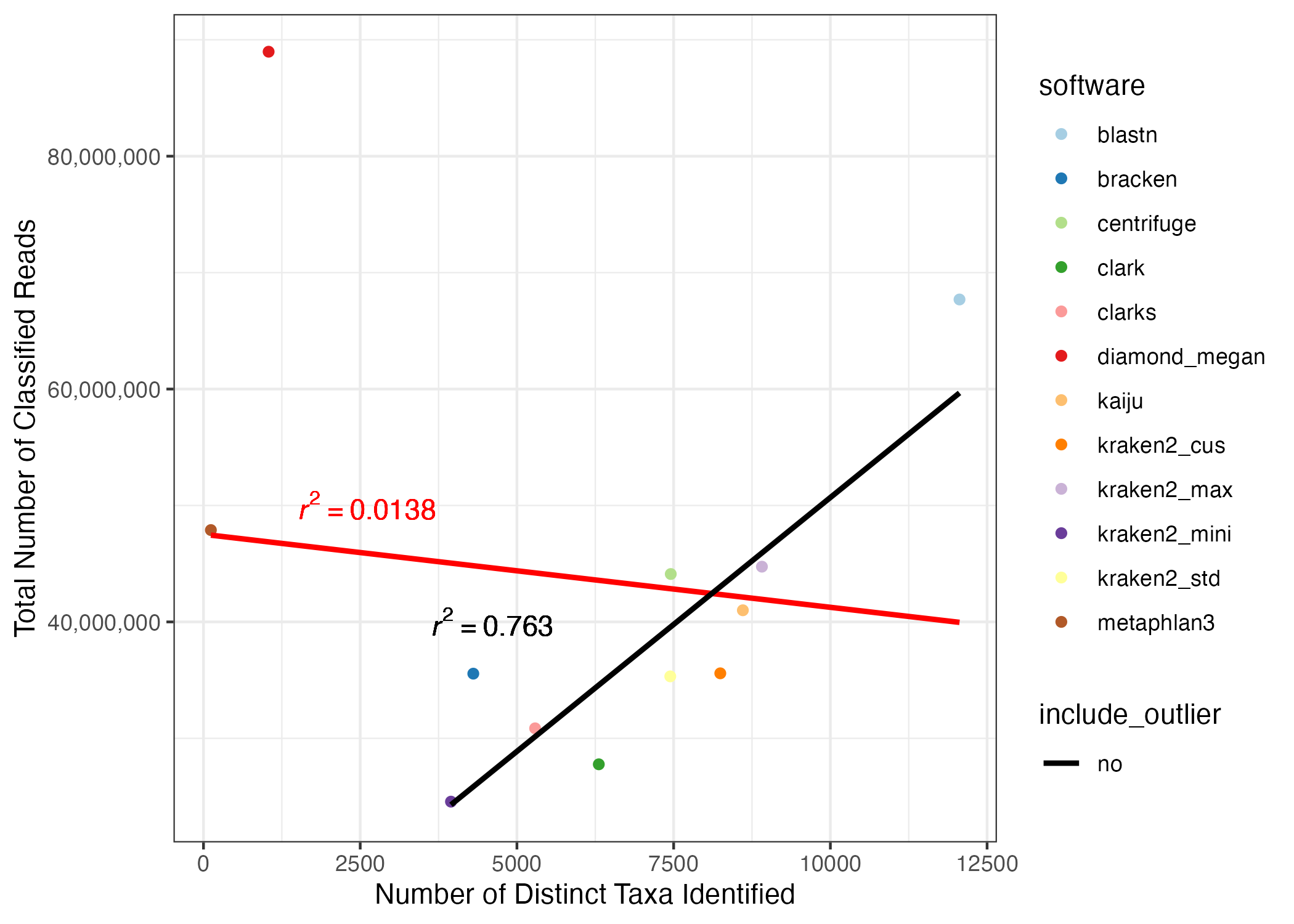
**

**Fig S2**. Total number of absolute reads classified under eukaryotic, bacterial, viral, and archaeal taxa by profiles of different software and DBs. Profiles with significant differences are shown in the figure with padj < 0.05. The red arrow at beginning of each significance bar located above the boxplot is the pivot profile of the comparisons represented by the specific bar, which means all the profiles pointed by the black ticks on the same bar has significantly different relationships with the pivot profile the red arrow is pointing to.


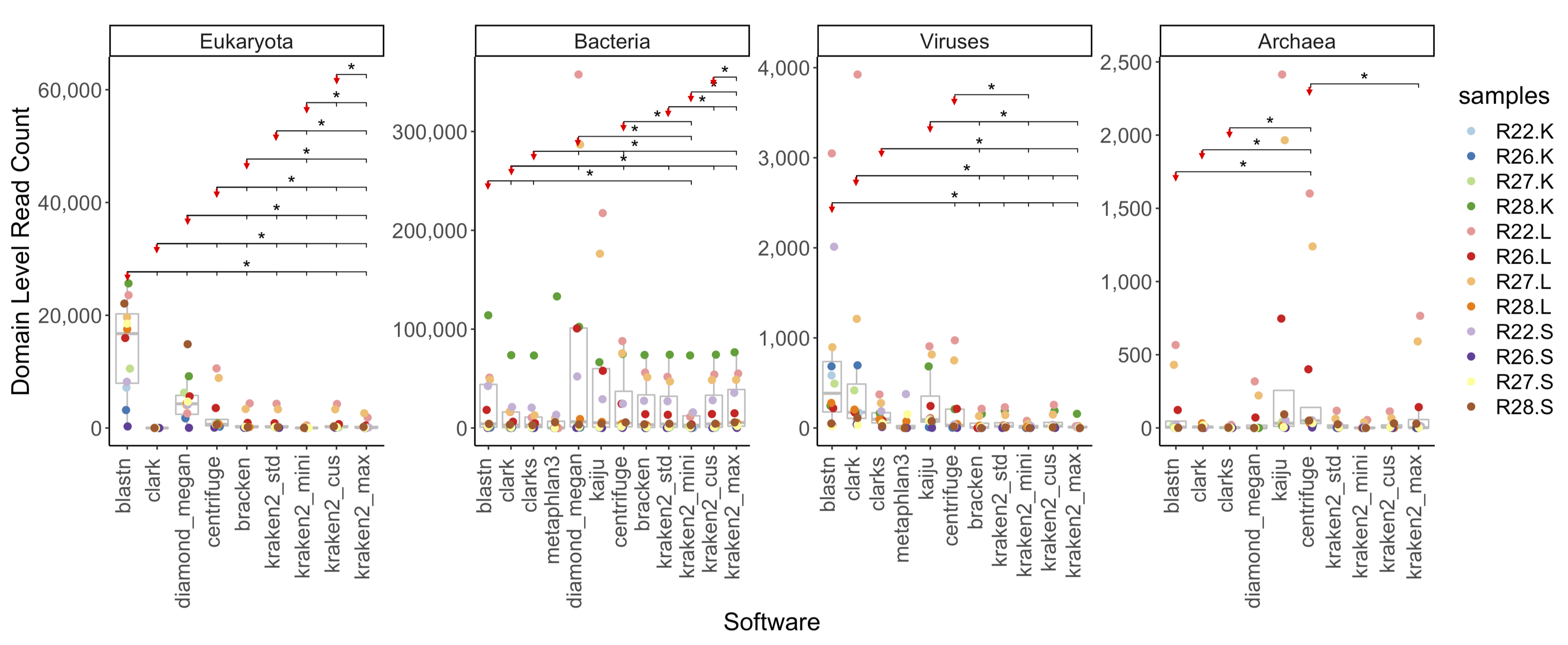


**Fig S3.** Relative precision rates for the distinct microbial taxa identified at the species level between profiles identified by the different software and DBs. The boxplots on each side represent the relative precision rates across samples being compared (A *vs*. B), where relative precision rates of profiles A and B are presented on the left- and right-hand sides, respectively.

**
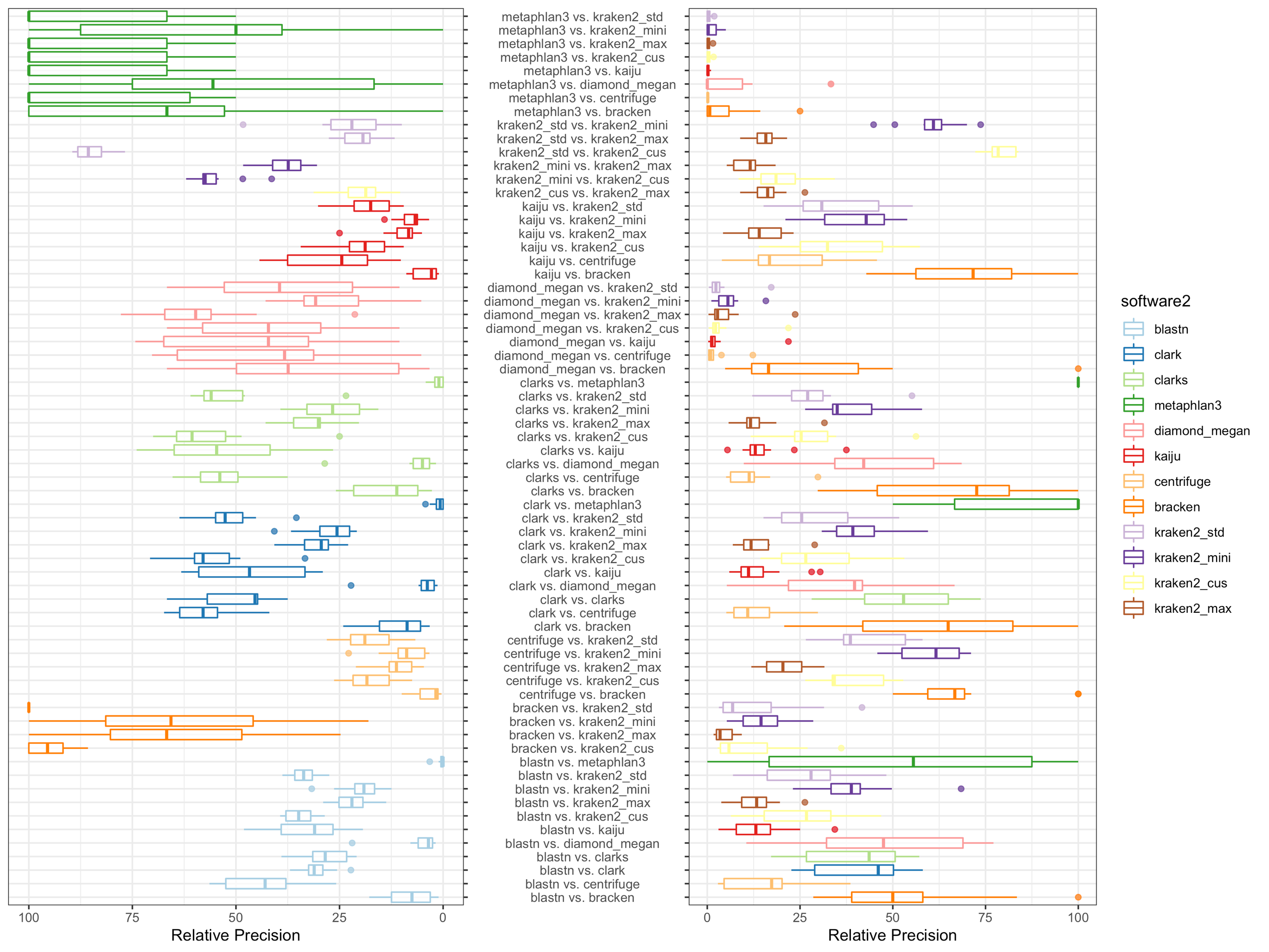
**

**Fig S4.** Comparisons in DA taxa identified between the lung and spleen samples in the dataset by different profiles. a) Distinct intersection between the DA taxa profiles identified at the species level. The bottom dot plot shows the identity of the profiles included in a set, the bar plot on top shows the size of the distinctly intersected DA taxa reported in the corresponding set in the dot plot, and the bar plot on the left shows the total number of DA species identified by each profile. b) Percentage of DA taxa identified by all profiles at each taxonomy level that are intersected between all software.


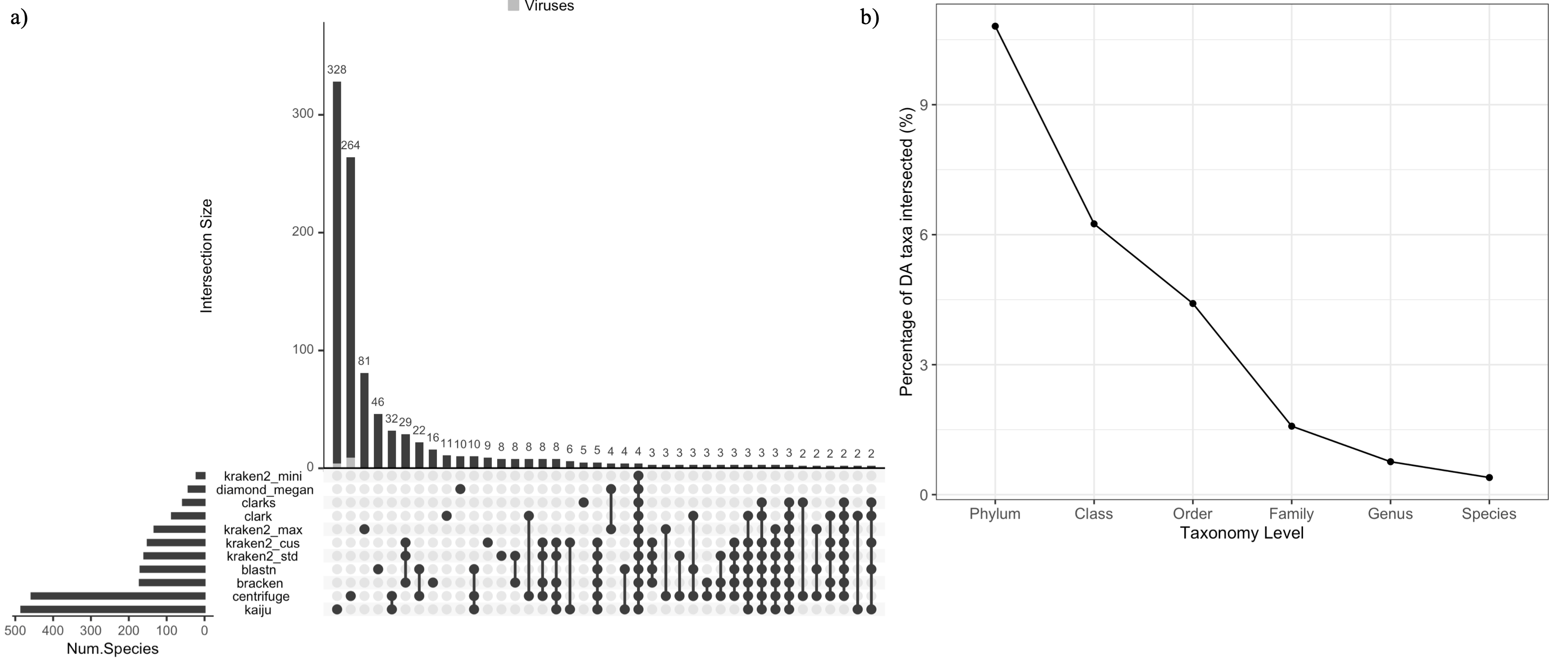


**Fig S5.** Comparisons in DA taxa identified between the kidney and spleen samples in the dataset by different profiles. a) Distinct intersection between the DA taxa profiles identified at the species level. The bottom dot plot shows the identity of the profiles included in a set, the bar plot on top shows the size of the distinctly intersected DA taxa reported in the corresponding set in the dot plot, and the bar plot on the left shows the total number of DA species identified by each profile. b) Percentage of DA taxa identified by all profiles at each taxonomy level that are intersected between all software.


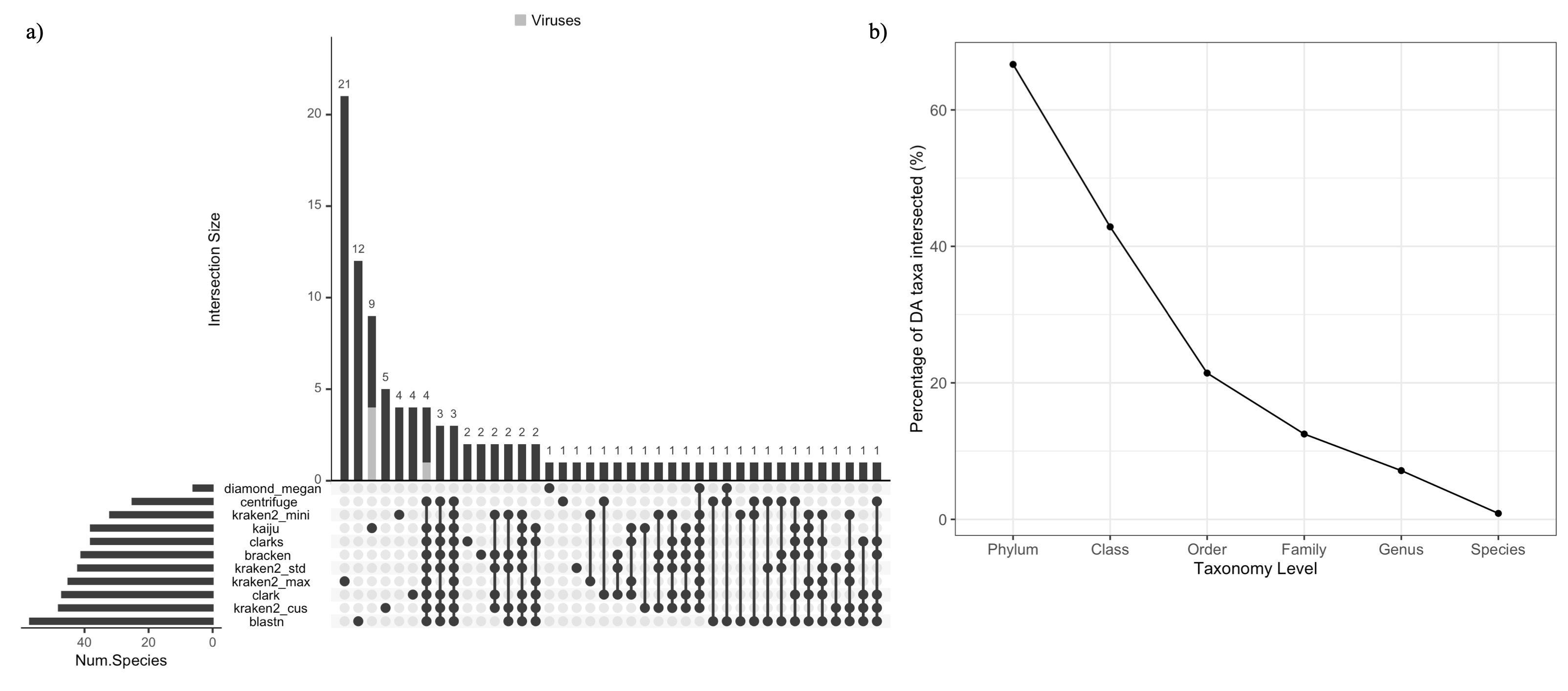

Supplement: S1 File — (DOCX) [file pone.0284031.s001.docx]
